# Antarctic Water Tracks: Microbial Community Responses to Variation in Soil Moisture, pH, and Salinity

**DOI:** 10.3389/fmicb.2021.616730

**Published:** 2021-01-27

**Authors:** Scott F. George, Noah Fierer, Joseph S. Levy, Byron Adams

**Affiliations:** ^1^Department of Biology, Brigham Young University, Provo, UT, United States; ^2^Department of Ecology and Evolutionary Biology and Cooperative Institute for Research in Environmental Sciences, University of Colorado Boulder, Boulder, CO, United States; ^3^Department of Geology, Colgate University, Hamilton, NY, United States; ^4^Monte L. Bean Museum, Brigham Young University, Provo, UT, United States

**Keywords:** Antarctica, Mars analog, water tracks, microbial ecology, extremophiles

## Abstract

Ice-free soils in the McMurdo Dry Valleys select for taxa able to cope with challenging environmental conditions, including extreme chemical water activity gradients, freeze-thaw cycling, desiccation, and solar radiation regimes. The low biotic complexity of Dry Valley soils makes them well suited to investigate environmental and spatial influences on bacterial community structure. Water tracks are annually wetted habitats in the cold-arid soils of Antarctica that form briefly each summer with moisture sourced from snow melt, ground ice thaw, and atmospheric deposition via deliquescence and vapor flow into brines. Compared to neighboring arid soils, water tracks are highly saline and relatively moist habitats. They represent a considerable area (∼5–10 km^2^) of the Dry Valley terrestrial ecosystem, an area that is expected to increase with ongoing climate change. The goal of this study was to determine how variation in the environmental conditions of water tracks influences the composition and diversity of microbial communities. We found significant differences in microbial community composition between on- and off-water track samples, and across two distinct locations. Of the tested environmental variables, soil salinity was the best predictor of community composition, with members of the *Bacteroidetes* phylum being relatively more abundant at higher salinities and the *Actinobacteria* phylum showing the opposite pattern. There was also a significant, inverse relationship between salinity and bacterial diversity. Our results suggest water track formation significantly alters dry soil microbial communities, likely influencing subsequent ecosystem functioning. We highlight how Dry Valley water tracks could be a useful model system for understanding the potential habitability of transiently wetted environments found on the surface of Mars.

## Introduction

The abiotic extremes characteristic of the McMurdo Dry Valleys (MDV) region (77–78° S, 160–164° E) (Fountain et al., 1999) select for particular taxa able to cope with the unique environmental conditions (Cary et al., 2010; Doran et al., 2010). The MDV region is a polar desert ecosystem with average air temperatures of −18°C, winter lows of −65°C, and summer temperatures that fluctuate around 0°C (Doran et al., 2002). Annual precipitation is limited to 3–50 mm (water equivalent) of snow (Fountain et al., 2010), most of which sublimates before entering a liquid phase (Chinn, 1993), making a vast majority of the terrestrial habitat highly arid. Fast, dry drainage winds descend from the polar plateau with speeds up to 37 m s^–1^, warming valley floors and lowering relative humidity, resulting in further desiccation of surface soils (Nylen et al., 2004). Soil salinity is extremely variable in coastal MDV, with solute salt concentrations ranging from 0 to 6000 eq m^–2^ from young coastal lowland surfaces to ancient glacial tills further inland (Toner et al., 2013). Biota within this region are also subject to dynamic solar radiation regimes, including 4 months of near or constant darkness (Dana et al., 1998).

The MDV lack vertebrates and vascular plants, with photosynthetic primary productivity limited to patchy distributions of cyanobacteria and algae found in lakes (Vincent et al., 1993), seasonally wetted streams and soils (Hawes and Howard-Williams, 2013; Stanish et al., 2013; Niederberger et al., 2015), and lithic niches (Friedmann and Ocampo, 1976; Friedmann, 1982; Pointing et al., 2009). The low complexity of this desert ecosystem makes it a well-suited natural system to investigate physical and chemical controls on soil microbial communities, with generalized findings that can be applied to other habitats (Van Horn et al., 2013).

Liquid water availability limits biological activity within this habitat where water availability is primarily regulated by low temperatures and limited net snow accumulation. During austral spring and summer, a hydrological continuum forms in MDV soils as differentially warmed soils and spatially heterogeneous snow and ice reservoirs generate different volumes of transient liquid water (Levy, 2015). These wetted soil areas in the cold desert range from those that are spatially extensive and perennial, such as glacier-fed streams and lakes, to those that are meter-scale and episodic and include hyporheic zones (i.e., wetted soils around and under lakes, ponds, and streams) (McKnight et al., 2007; Niederberger et al., 2015; Lee et al., 2018), water tracks (channelized subsurface groundwater flow) (Levy et al., 2011, 2013; Gooseff et al., 2013), and “wet patches” that form via deliquescence when exposed hygroscopic salts in the soil absorb atmospheric water vapor and generate liquid solutions (Seinfeld and Pandis, 2006; Levy et al., 2012).

Remote sensing data suggest that transiently wetted soils, specifically those removed from surface water bodies, occupy ∼5–10 km^2^ of the landscape during the seasonal thaw (Langford et al., 2015), a small but significant area that is projected to increase within this region due to ongoing climate change (Fountain et al., 2014). Previous work on water track physical, chemical, and biological processes suggest that water track soils are wetter, saltier, finer-grained, and more organic-matter-rich than adjacent, off-track soils (Levy et al., 2013; Ball and Levy, 2015).

At the surface, Antarctic water tracks are identified as visibly damp (dark) soils which grow downslope each summer with the release of meltwater from snow, glaciers, and ground ice (Harris et al., 2007; Levy et al., 2011). Water track liquids flow within the active layer beneath the soil surface but above the ice table, which is located above permanently frozen soil (Levy et al., 2011). Water track soil moisture and soil salinity are generally 5–10 times higher than other arid desert soils in the Dry Valleys (Gooseff et al., 2013; Levy et al., 2013). Accordingly, water tracks represent a pronounced alteration to the desert ecosystem and their presence may result in important shifts in soil microbial community composition, diversity, and ecosystem functioning.

Water tracks have been proposed as useful model systems for the episodically formed features on Mars termed recurring slope lineae (RSL) (Levy, 2012), which, like water tracks, are dark-toned, meter-scale features that grow downslope for hundreds of meters during seasonal warm periods (McEwen et al., 2011; Stillman et al., 2017). Among other similar characteristics, water tracks possess hygroscopic salts capable of deliquescence (Levy et al., 2011; Gooseff et al., 2013), which may contribute additional soil moisture beyond that derived from meltwater sources alone. Deliquescence has been identified as a mechanism for plausible transient water formations on Mars (Rennó et al., 2009; Smith et al., 2009; Martín-Torres et al., 2015), including RSL (Ojha et al., 2015). However, the role, quantity, or even presence of water in RSL has been challenged (Edwards and Piqueux, 2016; Dundas et al., 2017; Schmidt et al., 2017).

McMurdo Dry Valleys water tracks may be more analogous to transiently wetted habitats found historically on Mars. Over the last ∼3.5 billion years, it appears that surface environments on Mars experienced a series of climate successions from cold and semiarid to ultimately a hyperarid state (e.g., Carr and Head, 2010; Mahaffy et al., 2013). These climatic shifts on Mars would have had a significant impact on the evolution of an early Martian biosphere (Davila and Schulze-Makuch, 2016). It is plausible that early in Mars’ history (∼3.5 billion years ago), transiently wetted environments similar to Antarctic water tracks existed at the margins of highlands glaciated regions (Wordsworth, 2016), and if so, they would have represented important niches within the increasingly cold and arid planet.

Previous biological investigations of the Dry Valley water tracks are limited in number and have yet to produce concordant conclusions. In some cases, water track soil moisture enhancement has shown to elevate levels of microbial biomass relative to neighboring dry soils (Van Horn et al., 2013; Chan-Yam et al., 2019), though this increase has not always been observed (Ball and Levy, 2015). Other work suggests water tracks promote microbial activity, as measured by phosphorus depletion (Gooseff et al., 2013), CO_2_ fluxes (Ball and Levy, 2015), and *in vitro* microcosm radio-respiration assays (Chan-Yam et al., 2019). However, water track fluids are also highly saline, creating “dead zones” in the polar desert that limit habitability to only the most halotolerant organisms (Ball and Levy, 2015). Isolated “wet patches” can be so saline as to exceed water activity limits for cellular growth and reproduction (Levy et al., 2012; Rummel et al., 2014). An investigation of nematodes, the most abundant metazoan in the Dry Valleys, showed pronounced declines in population numbers when measured within water track soils (Gooseff et al., 2013). Several studies have found microbial communities in transiently wetted soils and water tracks have significantly different compositions compared to neighboring arid soils (Van Horn et al., 2014; Niederberger et al., 2015; Lee et al., 2018), although these differences has not always been observed (Chan-Yam et al., 2019).

One possible confounding factor that may have affected previous studies is differences in soil age and accumulated soil salinity. In an effort to mitigate this effect, we examined sediment profiles from two proximal locations with similar microclimate conditions, but different soil ages. The Goldman Glacier Basin (GB) water track flows through older, Taylor IV tills (2.1–37 Ma), while Water Track 1 (WT1) in the Lake Hoare basin flows through younger mixed Taylor III/Bonney tills (74–210 ka) (Bockheim et al., 2008).

We set out to identify how variation in the measured physical and chemical environment of water tracks within these two water track systems influence soil microbial community structure and diversity. A total of twenty samples from on- and off-track soils were analyzed to assess abiotic influences on microbial composition and diversity. Soil bacterial biodiversity was assessed using cultivation independent 16S rRNA gene sequencing.

We hypothesized that soil salinity, moisture, and pH are significantly different within water tracks than outside of them, and that these geochemical factors are significantly correlated with microbial community structure and diversity. We also hypothesized that soil microbial community composition is significantly different between on-track and off-track habitats, and between the two tested water track systems in Taylor Valley. Namely, the Goldman Glacier Basin water track, and that of the Lake Hoare Basin, Water Track 1. We further hypothesized that the seasonal formation of water tracks results in significantly higher community diversity, richness, and evenness compared to off-track habitats due to increased water availability. Finally, we hypothesized that soil position (i.e., on-track and off-track), salinity, moisture, pH, and the two different site locations of water track systems are significant explanatory variables for soil microbial community composition.

## Materials and Methods

### Sampling and Soil Geochemistry

Soil samples and pore water were collected from the Lake Hoare and Goldman Glacier Basins of Taylor Valley, Antarctica during the austral summer of 2012–2013. Two separate water track systems within Taylor Valley were selected for analysis, Water Track 1 (77.64°S, 162.94°E) and Goldman Glacier Basin (77.67°S, 162.93°E), which are approximately 3.3 km apart. Sediment and pore water collected from the darkened portions of water tracks are designated here as “on-track,” and samples from the adjacent lighter soils are classified as “off-track.” Off-track samples were located at least 5 m from the current edge of the water track (Levy et al., 2013). Wet, on-track soils have a typical albedo of 0.15, while the off-track soil albedo is generally 0.22 (Levy et al., 2013), making them readily distinguishable in the field. Samples were collected from the upper 10 cm of the soil horizon using aseptic techniques and were stored in sterile Whirl-Pak bags at −20°C until processing. Latitude and longitude of the collected samples, as well as sample states (i.e., on-track or off-track), were recorded at the time of collection (see Table 1 for details).

**TABLE 1:** Summary of sample abiotic properties.

**Sample**	**On-/off-water track**	**System**	**EC (dS/m)**	**GWC (%)**	**pH**	**Latitude**	**Longitude**
1	Off	WT1	0.02	2.03	7.99	−77.6393	162.9406
2	Off	WT1	0.03	2.07	8.43	−77.6428	162.9303
3	Off	GB	0.00	2.48	8.49	−77.6669	162.9250
4	On	GB	0.19	3.47	8.30	−77.6668	162.9258
5	Off	WT1	0.01	1.31	8.33	−77.6326	162.9340
6	On	WT1	0.08	7.57	8.24	−77.6427	162.9300
7	On	WT1	0.05	3.79	8.34	−77.6458	162.9177
8	Off	GB	0.05	1.00	8.40	−77.6690	162.9262
9	On	WT1	0.16	1.51	8.31	−77.6364	162.9354
10	On	WT1	0.07	0.79	8.41	−77.6392	162.9404
11	On	GB	0.47	4.35	8.35	−77.6720	162.9325
12	Off	WT1	0.01	2.63	8.84	−77.6460	162.9182
13	On	WT1	0.05	2.84	8.06	−77.6345	162.9351
14	Off	WT1	0.01	1.66	8.02	−77.6363	162.9359
15	On	GB	0.05	4.98	7.96	−77.6690	162.9256
16	On	GB	0.73	2.18	8.00	−77.6644	162.9253
17	On	WT1	0.25	3.81	8.13	−77.6364	162.9353
18	Off	WT1	0.01	1.31	8.37	−77.6326	162.9340
19	Off	GB	0.01	2.02	–	−77.6721	162.9322
20	Off	GB	0.09	2.34	7.99	−77.6644	162.9266

Gravimetric water content (GWC) was calculated to obtain soil moisture values for each sample. Electrical conductivity (EC), a proxy of salinity, was also measured for each sample. GWC was calculated as the percentage of dry weight of sediment per sample, drying approximately 100 g of soil at 105°C for 24 hours and then reweighing the sample. EC (dS/m) was obtained for each sample using a Decagon Devices 5TE probe. Soil pH was measured in a 1:2 0.01 M CaCl_2_ solution (Thermo Orion pH Meter Model 410A+, Thermo Electron, Waltham, MA, United States).

### DNA Extraction and Taxonomic Profiling

DNA extraction and microbial community analyses were conducted using the cultivation-independent 16S rRNA gene sequencing approach as described in Prober et al. (2015). Total genomic DNA was extracted from each soil sample using the MO BIO’s PowerSoil DNA Isolation Kit (MO BIO Laboratories Inc., Carlsbad, CA, United States). For microbial analyses, the V4 hypervariable region of the 16S rRNA gene was PCR amplified using the 515f and 806r primer pair which captures both Bacteria and Archaea. Three PCRs were run per sample, with the amplicons from the replicate reactions pooled. Each primer pair included Illumina adapters and 12-bp error-correcting barcodes unique to each sample (Thompson et al., 2017). After gel visualization to confirm amplification, we used a PicoGreen dsDNA assay to quantify amplicon yields, with the amplicons then pooled together in equimolar concentrations for sequencing on the Illumina MiSeq instrument. DNA sequencing was completed at the University of Colorado Next Generation Sequencing Facility using the 2 × 150 bp paired-end sequencing chemistry. Four DNA extraction and four no-template PCR “blanks” were included in the run to check for potential contamination.

Sequences were demultiplexed using a custom Python script (‘prep_fastq_for_uparse.py’^1^), with the UPARSE pipeline used for quality filtering and phylotype (i.e., operational taxonomic unit) clustering (Edgar, 2013). Quality filtering was conducted using a maximum e-value of 0.5 with paired-end sequences merged prior to downstream processing. Representative sequences from returned phylotypes that were not ≥75% similar to sequences contained in the Greengenes database (McDonald et al., 2012) were removed; afterward, the raw sequences were mapped to phylotypes at a 97% similarity cutoff. Taxonomic classification of each phylotype was determined using the Ribosomal Database Project classifier (Wang et al., 2007) against the Greengenes database with a confidence threshold of 0.5.

### Statistical Analyses

**FIGURE 1:**
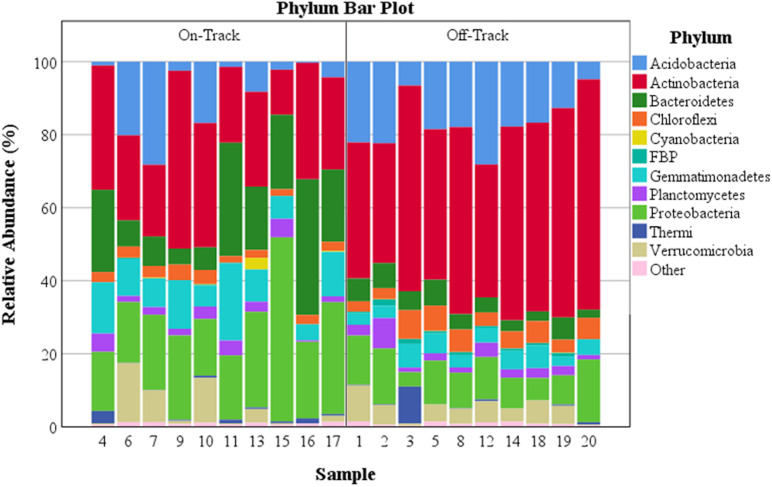
The percent relative abundance of major soil bacterial taxa across all samples. Phyla with a relative abundance >0.39% either on or off water tracks are identified.

To determine soil chemical and physical effects on community structure, statistical tests of inter- and intra-site relationships and differences were undertaken. Accordingly, phylotype data were rarefied in R (R Core Team, 2017) to 11,649 reads per sample with the vegan package (Oksanen et al., 2019) before all diversity analyses. Alpha diversity metrics were calculated using the microbiome package (Lahti and Shetty, 2012), for Pielou’s evenness, and the vegan package (Oksanen et al., 2019), for Shannon diversity (H’) (Supplementary Table S1). Independent *t*-tests of the alpha diversity metrics between on and off-track samples were run in SPSS (IBM, 2016). Phyla relative abundances (Figure 1 and Supplementary Table S2) were calculated with the funrar package (Grenié et al., 2017). Stacked histograms and clustered box plots for phyla relative abundances were generated with SPSS (IBM, 2016).

To test for correlations between community composition and measured soil properties, Mantel tests based on Pearson’s product-moment correlations were run for each variable using the vegan package (Oksanen et al., 2019). Hypotheses regarding relationships between soil properties and microbial diversity, richness, and evenness were also explored using Pearson correlation tests. Mann–Whitney U and independent *t*-tests, as appropriate, were used to test significant differences in soil chemical and physical properties between on- and off-track samples.

PERMANOVA tests, with pairwise distances calculated using Bray–Curtis distance, were run in the vegan package (Oksanen et al., 2019) to test for significant differences in soil microbial communities between on-track and off-track samples; WTI and GB systems; on-track communities of GB and on-track communities of WT1; and finally, off-track communities of GB and off-track communities of WT1. A multidimensional scaling (MDS) plot informed by a Bray–Curtis dissimilarity matrix was created in the vegan package (Oksanen et al., 2019) to represent microbial community clustering based on sample type (On/Off-track) and water track system (GB/WT1) within the axes of soil salinity, moisture, and pH.

Redundancy analysis (RDA) with variation partitioning was run using the vegan package (Oksanen et al., 2019) to estimate the percent at which the measured abiotic variables, individually and collectively, explained microbial community composition in the tested soils. ANOVA tests were run on the specific canonical axes produced from the underlying RDAs to see if the explanatory variables were significant in the partition variance.

## Results

### Soil Properties

The mean on-track soil moisture of the Lake Hoare Basin water tracks was significantly higher than the adjacent off-track soils (*P* = 0.03). Water track soil moisture was, on average, ∼1.9 times higher than neighboring dry soil (3.53–1.89%), ranging from 0.79–7.57% on-track, and 1.00–2.63% off-track (Table 1).

On-track soil values were also significantly more saline (*U* = 93.5, *P* < 0.001) than the proximal off-track counterparts. Median salinity on-track was ten times higher than off-track (0.12 dS/m compared to 0.01 dS/m). The range of on-track salinities were more variable (0.05 dS/m to 0.73 dS/m) than off-track salinities, which were consistently low across the tested samples (0.00 dS/m to 0.09 dS/m).

Soil pH was fairly uniform across all samples, with no significant difference in the mean pH between on-track and off-track soils (8.21 and 8.32, respectively) (*P* = 0.31) (Table 1). There was no apparent correlation between any of the measured abiotic variables, namely: moisture and pH [*r*(19) = −0.16, *P* = 0.52], pH and salinity [*r*(19) = −0.25, *P* = 0.30], and salinity and moisture [*r*(20) = 0.52, *P* = 0.15].

### Microbial Communities

Across all samples, we detected a total of 1457 unique phylotypes after rarefaction, with the number of phylotypes per sample ranging from 182 to 713 (Supplementary Table S1). Eight archaeal phylotypes, not included in analyses here, were identified as belonging to two phyla, and represented <0.23% of the sequences. On-track samples had higher average numbers of phylotypes (420 phylotypes per sample ±56) compared to off-track samples (392 ± 32), though this was not significant (*P* > 0.7). There was also no significant difference in average Shannon diversity (*P* > 0.4) between on-track (4.13 ± 0.23) and off-track (4.37 ± 0.12) samples. Pielou’s evenness was not significantly different on- or off-track (*P* > 0.1), although it was higher off-track than on-track (0.74 ± 0.01, 0.69 ± 0.03).

**FIGURE 2:**
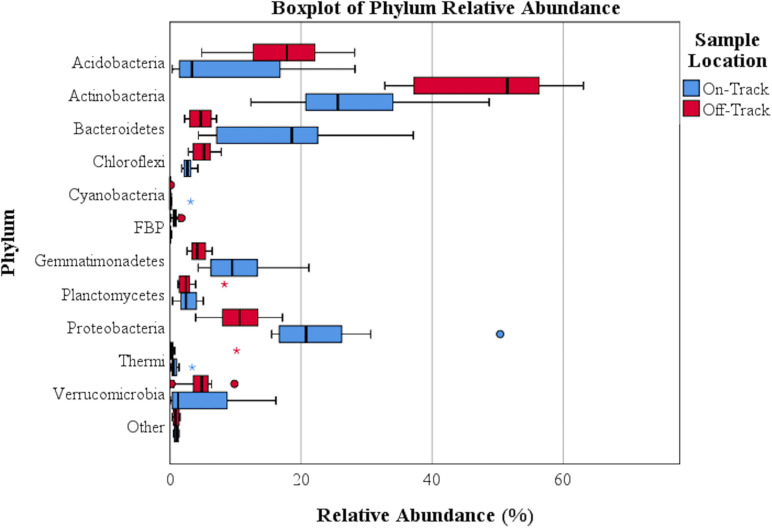
A boxplot of bacterial relative abundance on and off the tested water tracks. Asterisks represent extreme outliers; solid circles represent standard outliers. Phyla with a relative abundance >0.39% either on or off water tracks are identified.

The majority of recovered microbial communities were comprised of a handful of phyla (Figure 1 and Supplementary Table S2), particularly *Actinobacteri*a, which was the most abundant phylum detected in soils both on and off water tracks (27.6% and 48% of reads, respectively). Other abundant phyla on and off water tracks included *Proteobacteria* (23.8%, 10.6%), *Bacteroidetes* (17.4%, 4.8%) *Gemmatimonadetes* (10.4%, 4.4%) and *Acidobacteria* (8.5%, 16.8%). The relative abundance of these five phyla, from the 25 phyla identified, accounted for 87.7% of all communities on-track and 84.5% of those off-track (Supplementary Table S2). For some phyla, the intra-sample (e.g., on-track versus on-track) variation in relative abundances was considerable (Figure 2).

**FIGURE 3:**
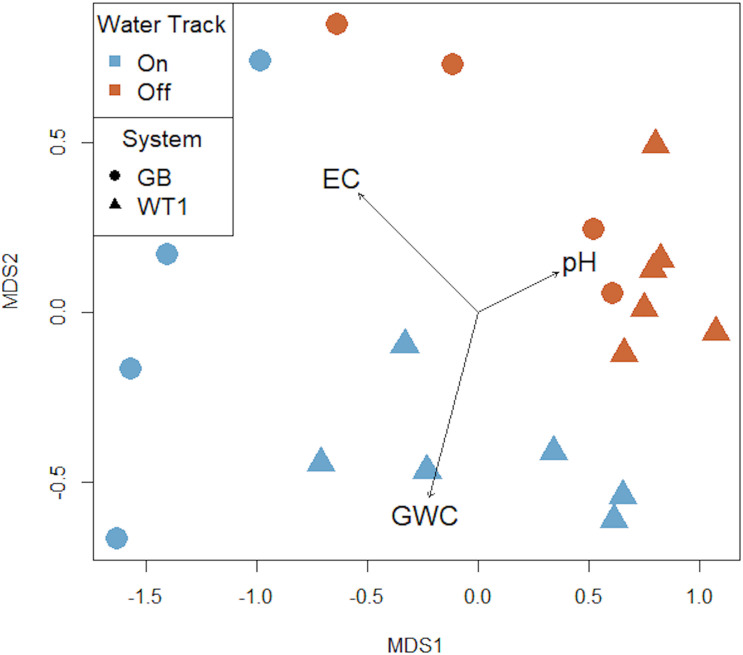
Multidimensional scaling (MDS) plot of soil microbial community composition. Water tracks (blue) harbor significantly different bacterial communities from those collected off-track (red) (PERMANOVA *R*^2^ = 0.203, *P* = 0.001). Other significant differences include water track system, namely the Goldman Glacier Basin (GB) and Water Track 1 (WT1) (PERMANOVA *R*^2^ = 0.156, *P* = 0.004); on- versus on-track soil communities between GB and WT1 (PERMANOVA *R*^2^ = 0.33, *P* = 0.01); and off- versus off-track soils communities between GB and WT1 (PERMANOVA *R*^2^ = 0.208, *P* = 0.033). The black vectors represent measured environmental parameters (pH, EC = electrical conductivity, GWC = gravimetric water content) that fit into the ordination space. *R*^2^ = 0.995. Stress score = 0.068 in two dimensions.

We observed a high degree of variation in the composition of bacterial communities, with community composition patterns correlated with select physiochemical properties and spatial scale (Figure 3). On-track microbial communities were significantly different than those off-track (PERMANOVA *R*^2^ = 0.203, *P* < 0.001). Microbial communities between the GB and WTI water track systems were also significantly different (PERMANOVA *R*^2^ = 0.156, *P* = 0.004). Finally, comparisons of intra-track types between systems, namely on-track to on-track and off-track to off-track soils between GB and WT1, showed significant differences in microbial communities (PERMANOVA *R*^2^ = 0.33, *P* = 0.01, PERMANOVA *R*^2^ = 0.208, *P* = 0.03, respectively). On-track communities in the MDS plot were distinctly clustered with each other, as were off-track communities and assemblages based on system (GB/WT1) (Figure 3).

A Mantel statistic based on Pearson’s correlation showed a statistically significant relationship between soil salinity and community composition [*r*(20) = 0.41, *P* = 0.005]. However, a Mantel test exploring possible correlations between soil moisture and microbial community composition was not significant [*r*(20) = 0.20, *P* = 0.065], as was the relationship between pH and community composition [*r*(19) = −0.04, *P* = 0.58]. Salinity was the only environmental variable that was significantly correlated with microbial diversity (H’) [*r*(20) = −0.537, *P* = 0.02], richness [*r*(20) = −0.45, *P* = 0.045], and evenness (H’) [*r*(20) = −0.48, *P* = 0.03]. In all instances, as salinity increased these diversity metrics declined.

Redundancy analysis with variance partitioning showed highly similar patterns with the Mantel tests. Specifically, RDA with variance partitioning estimated that collectively, sample location in regard to water tracks (On/Off-Track), soil salinity, and the water track system location (WT1/GB) were significant explanatory variables for 22.7% of the tested soils’ microbial community composition (ANOVA, *P* = 0.002). Sample location (On/Off-Track) explained 5.8% of the variation in community composition conditioned on the other variables included in the model (ANOVA, *P* = 0.03). For soil salinity, this was 7.7% (ANOVA, *P* = 0.046), and for system location, it was 6.7% (ANOVA, *P* = 0.02). The canonical axes of soil moisture and pH were not significant explanatory variables in structuring the tested microbial communities (ANOVA, *P* = 0.38, *P* = 0.58, respectively).

## Discussion

The annual formation of water tracks in this polar desert ecosystem represents a significant alteration in this relatively low-complexity terrestrial landscape. Similar to other observations (Gooseff et al., 2013; Levy et al., 2013), our tested average water track salinity was an order of magnitude and significantly higher than adjacent ‘non-water track’ soils. The observed differences in community composition were most strongly associated with differences in soil salinity (Figure 3), a pattern that is in agreement with previous studies (Lee et al., 2012; Van Horn et al., 2013). As salinity increased, the relative abundance of *Bacteroidetes* increased considerably, with observed decreases in *Actinobacteria*, suggesting a shift toward a more halotolerant community. No correlations were found among soil salinity, pH, and moisture.

We observed no significant differences in average diversity, richness, or evenness between on-track and off-track soils, in spite of nearly twofold increases in soil moisture and tenfold increases in salinity when on track. However, soil salinity was significantly correlated with diversity, richness and evenness, and in all cases the relationship was negative. Similar significant soil salinity relationships with microbial diversity (Zeglin et al., 2011; Van Horn et al., 2014) have been observed elsewhere in the Dry Valleys. Salinity measured by Zeglin et al. (2011) was largely within the range measured in our study. Salinity measured by Van Horn et al. (2014) reached levels several orders of magnitude above ours, yet the same relationship was exhibited. Our findings suggest that MDV soil microbial richness is sensitive to even moderate changes in salinity, with higher soil salinities associated with less diverse bacterial communities.

Soil salinity can have a pronounced direct and indirect affect in shaping microbial communities and their ecological responses (Rietz and Haynes, 2003; Lozupone and Knight, 2007; Rath and Rousk, 2015; O’Brien et al., 2019; Rath et al., 2019). Salt-affected soils generally show decreases in microbial respiration (Sardinha et al., 2003; Yuan et al., 2007), biomass (Rietz and Haynes, 2003; Yuan et al., 2007), and extracellular enzymatic activity (Rietz and Haynes, 2003; Ghollarata and Raiesi, 2007). In more extreme cases, as demonstrated in aquatic habitats, elevated salt concentrations can prevent metabolic activity (Oren, 2013) and inhibit life via denaturing of biological macromolecules (Hallsworth et al., 2007). However, microbial communities within salt-affected areas should exhibit adaptive and taxonomic responses if the salinity is elevated to meaningful ecological levels (Rath and Rousk, 2015; Gunde-Cimerman et al., 2018; O’Brien et al., 2019), which may alter at least some ecosystem functions (Kimbrel et al., 2018; Rath et al., 2019).

The absence we observed of statistically significant correlations between water content and community composition in the MDV was also observed in several, though not all, sites sampled by Van Horn et al., 2013. Chan-Yam et al. (2019) similarly found no significant correlation between community composition and soil moisture content from their MDV soil investigations. However, our MDS plot strongly hints that water content, which can be seasonally dynamic, may play an important role in community structure.

Soil pH has been shown to have a strong influence on bacterial community structure at the continental scale (Fierer and Jackson, 2006; Lauber et al., 2009). Regional analyses looking at the influence of soil pH on microbial communities in Antarctica are in agreement with these large-scale studies (Smith et al., 2010; Van Horn et al., 2013), as are some localized studies (Van Horn et al., 2013; Chan-Yam et al., 2019). However, other localized Dry Valley investigations failed to find soil pH as a significant factor in community partitioning (Lee et al., 2018), or being correlated with diversity metrics (Zeglin et al., 2011). Soil pH from our study was reasonably constrained across all collected samples (Table 1), which may be one reason we did not identify significant relationships between it and microbial community composition, diversity, richness or evenness.

The off-track samples had communities dominated by the phyla *Acidobacteria, Actinobacteria, Proteobacteria*, and to a lesser extent *Bacteroidetes, Chloroflexi, Gemmatimonadetes, Verrucomicrobia*, and *Planctomycetes* (Figure 1 and Supplementary Table S2). The dominance of these particular phyla within arid MDV soil communities are concordant with previous studies, especially for the phyla *Acidobacteria* and *Actinobacteria* (Pointing et al., 2009; Zeglin et al., 2011; Lee et al., 2012; Niederberger et al., 2015). The phyla *Acidobacteria* and *Actinobacteria* have also been found to be among the most abundant taxa in the extremely arid and significantly warmer soils of the Atacama Desert in Chile (Crits-Christoph et al., 2013).

On-track soil samples were composed largely of the phyla *Acidobacteria, Actinobacteria, Bacteroidetes, Gemmatimonadetes*, and *Proteobacteria*. *Acidobacteria* and *Actinobacteria* were the two most dominant phyla off-track, but their abundances dropped nearly in half within water track samples (Figure 1 and Supplementary Table S2). *Bacteroidetes, Gemmatimonadetes*, and *Proteobacteria* saw dramatic increases in their relative abundances within water track soils.

Other investigations regarding phyla in transiently wetted Dry Valley soils observed similar trends, with the phyla *Proteobacteria* (Stanish et al., 2013; Niederberger et al., 2015) and *Bacteroidetes* (Zeglin et al., 2011) as the dominant members in wetter soils.

Several genera were found to be closely related to known extremophilic and extremotolerant taxa. Members from the genus *Gillisia* (phylum Bacteroidetes) were notably abundant in on-track samples. Closely related psychrophilic isolates from this genus were also found in Antarctic Lake Fryxell of Taylor Valley (Van Trappen et al., 2004), within soils from an Antarctic valley further south (Niederberger et al., 2015), and in Antarctic maritime environments (Bowman and Nichols, 2005). Phylotypes from the genus *Rubrobacter* (phylum Actinobacteria) were present in every sample, a genus which includes isolates which have been shown to exhibit resistance to ionizing radiation (Rainey et al., 2005) (Ferreira et al., 1999).

The genus *Modestobacter* (phylum Actinobacteria) was also detected, which has been isolated from the hyperarid Atacama Desert soils of Chile (Busarakam et al., 2016) and from the nearby Asgard Range of Antarctica (Mevs et al., 2000). A phylotype from genus *Truepera* (phylum Thermi) was also characterized. *Truepera* has been found in the ephemeral Lake Lucero playa of New Mexico, United States (Sirisena et al., 2018), an episodically wetted environment which shifts between a freshwater habitat and a hypersaline dry desert. Isolates from the genus *Truepera* have also shown to be highly resistant to ionizing radiation (Albuquerque et al., 2005). *Pseudoxanthomonas* and *Sphingomonas* (Phylum Proteobacteria) were present, genera which have been cultured from both saline and freshwater lakes in the Transantarctic Mountains and Shackleton Range of Antarctica (Peeters et al., 2011). The identification of phylotypes closely related to known psychrophilic, halotolerant, and ionizing-radiation-resistant isolates suggests, though does not confirm, adaptations found within the sampled microbial communities.

Other possible explanatory variables in shaping MDV soil microbial structure and diversity are the legacy influences associated with long-term water track presence. Within our study at least one water track system, Water Track 1, has persisted in a remarkably similar form and shape since at least 1911. This valuable information was preserved by photographic evidence gathered during Robert Falcon Scott’s *Terra Nova* Expedition, evidence which has been compared with present-day imagery (Levy et al., 2013). Temporal legacies associated with long-term water track presence may therefore be reasonably important in shaping microbial communities of water tracks, though this is challenging to test. A possible approach could include a time series investigation of newly forming water tracks that are now entering historically dry soils (Fountain et al., 2014).

Water tracks within the MDV region represent a small, though important, area of the cold-arid desert that is anticipated to expand with ongoing climate change. Our investigation found significant differences between microbial communities on- and off-water track samples and at different water track system locations. Of the tested variables, we found salinity to be the best predictor of microbial community composition, with *Bacteroidetes* concentrated at higher levels of salinity and *Actinobacteria* in low-saline soils. The microbial communities appeared to be sensitive to even moderate variations in salinity. Increases in salinity significantly correlated with decreases in microbial diversity, richness and evenness. There were no significant differences for microbial diversity, richness, or evenness on- and off-track. Soil moisture in this study was significantly higher within water track samples, yet it was not meaningfully correlated with community composition or diversity. Our research suggests this low complexity environment has complex abiotic and spatial influences upon microbial communities. Results from this study indicate water track formation significantly alters the arid soil microbial community composition in Antarctica soils, and therefore, possibly ecosystem functions. Water tracks may also serve as useful models for transiently wetted habitats that may have existed, or exist, on Mars surface.

## Data Availability Statement

The authors acknowledge that the data presented in this study must be deposited and made publicly available in an acceptable repository, prior to publication. Frontiers cannot accept a manuscript that does not adhere to our open data policies. The data presented in this study are deposited in the Environmental Data Initiative (EDI) Repository at https://doi.org/10.6073/pasta/a98c5ce00cc51d5424b07aebcfcf9f74 (George et al., 2020).

## Author Contributions

NF and JL conceived and designed the experiment. JL carried out the field work, with sample processing by JL and SG. SG analyzed the data. SG, NF, JL, and BA wrote the manuscript. All authors contributed to the article and approved the submitted version.

## Conflict of Interest

The authors declare that the research was conducted in the absence of any commercial or financial relationships that could be construed as a potential conflict of interest.

## Footnotes

1 https://github.com/leffj/helper-code-for-uparse
