# Supplementary material for: Antarctic Water Tracks: Microbial Community Responses to Variation in Soil Moisture, pH, and Salinity
Source: Front Microbiol. 2021 Jan 27;12:616730. doi: 10.3389/fmicb.2021.616730 (PMC7873294; doi:10.3389/fmicb.2021.616730)
Supplement: Supplementary file 1 [file Data_Sheet_1.docx]

SUPPLEMENTARY TABLE S1. BIODIVERSITY INDICES AND ESTIMATORS

| *Sample* | *On/Off Water Track* | *No. of OTU_0.03_* | *Shannon Diversity (H’)* | *Evenness* |
| --- | --- | --- | --- | --- |
| 1 | Off | 532 | 4.587406 | 0.7308694 |
| 2 | Off | 324 | 4.429969 | 0.7663320 |
| 3 | Off | 277 | 3.974549 | 0.7067099 |
| 4 | On | 257 | 3.838726 | 0.6917774 |
| 5 | Off | 456 | 4.809418 | 0.7855326 |
| 6 | On | 533 | 4.718525 | 0.7515344 |
| 7 | On | 573 | 4.872034 | 0.7671424 |
| 8 | Off | 461 | 4.576342 | 0.7461349 |
| 9 | On | 357 | 3.725152 | 0.6337733 |
| 10 | On | 713 | 5.030479 | 0.7657345 |
| 11 | On | 263 | 3.881792 | 0.6966413 |
| 12 | Off | 434 | 4.283944 | 0.7054031 |
| 13 | On | 566 | 4.686581 | 0.7393723 |
| 14 | Off | 439 | 4.746352 | 0.7800727 |
| 15 | On | 271 | 3.021878 | 0.5394170 |
| 16 | On | 182 | 3.06667 | 0.5892902 |
| 17 | On | 485 | 4.483447 | 0.7249902 |
| 18 | Off | 410 | 4.603957 | 0.7652655 |
| 19 | Off | 396 | 4.176301 | 0.6982130 |
| 20 | Off | 192 | 3.569026 | 0.6788453 |

**Supplementary Table S2.**

| **Relative Abundance (Mean % of Sequences)** | | |
| --- | --- | --- |
| **Phylum** | **On Water Track** | **Off Water Track** |
| Acidobacteria | 8.529 | 16.764 |
| Actinobacteria | 27.588 | 48.026 |
| Armatimonadetes | 0.053 | 0.274 |
| Bacteroidetes | 17.395 | 4.775 |
| BRC1 | 0.288 | 0.362 |
| Chlamydiae | 0.015 | 0.010 |
| Chlorobi | 0.218 | 0.137 |
| Chloroflexi | 2.797 | 5.039 |
| Cyanobacteria | 0.397 | 0.056 |
| Elusimicrobia | 0.014 | 0.009 |
| FBP | 0.094 | 0.795 |
| Fibrobacteres | 0.003 | 0.000 |
| Firmicutes | 0.046 | 0.014 |
| Fusobacteria | 0.000 | 0.002 |
| Gemmatimonadetes | 10.368 | 4.392 |
| MVP-21 | 0.003 | 0.053 |
| Nitrospirae | 0.161 | 0.019 |
| OD1 | 0.017 | 0.004 |
| Planctomycetes | 2.788 | 2.857 |
| Proteobacteria | 23.783 | 10.563 |
| Thermi | 0.826 | 1.228 |
| TM6 | 0.002 | 0.000 |
| TM7 | 0.152 | 0.036 |
| Verrucomicrobia | 4.457 | 4.579 |
| WPS-2 | 0.007 | 0.008 |
|  |  |  |

**Supplementary Table S2.** Changes in the relative abundance of all bacterial phyla between the tested on and off water track habitats. Darker colors in the heat map denote higher mean relative abundances.
